# Pattern of Tobacco Use among Children and Adolescents with comorbid Psychiatric Illnesses

**DOI:** 10.1192/j.eurpsy.2022.2152

**Published:** 2022-09-01

**Authors:** I. Afridi

**Affiliations:** CPSP (College of Physicians & Surgeons, Pakistan), Faculty Of Psychiatry, Karachi, Pakistan

## Abstract

**Introduction:**

Tobacco use is clearly harmful for mental as well as physical health especially among persons <18 yeards age. It is used in multiple forms in many countries such as, somoling chewing etc. It is impotant to know the pattern of tobacco use and the comorbid psychiatric illnesses inthis age group.

**Objectives:**

To identify the pattern of tobacco use among cases <18 years age with various psychiatric disorders.

**Methods:**

It was a cross-sectional study conducted at a psychiatric clinic at Karachi, on all consecutive cases <18 years. Diagnostic criteria of ICD-10 were used.

**Results:**

A total number of 700 consecutive cases below the age of 18 years reporting to psychiatric clinic were inducted in this study. Out of them 107 (15%) patients reported use of tobacco. Among them 83 (77% ) used pan with tobacco. The psychiatric illnesses identified were depressive disorder (39%) & conversion disorder (15%).

**Conclusions:**

Tobacco use (predominantly in the form of chewing), is common amongst children and adolescents reporting for psychiatric consultation. It is important to develop strategies at a community level with legal restriction/implementation selling tobacco to children. Moreover, psychiatric evaluation should be done in all children and adolescents identified as using tobacco, and the term “smoking cessation clinic” should be replaced with “tobacco cessation clinic/ services”.

**Disclosure:**

No significant relationships.

